# Rash induced by enteral vancomycin therapy in an older patient in a long-term care ventilator unit: case report and review of the literature

**DOI:** 10.1186/s13223-018-0293-2

**Published:** 2018-11-06

**Authors:** Jeremy Barron, Adolfo Lattes, Esther-Lee Marcus

**Affiliations:** 1 Chronic Ventilator Unit, Herzog Medical Center, Givat Shaul St, POB 3900, 91035 Jerusalem, Israel; 2 Department of Medicine, Johns Hopkins University, 5505 Hopkins Bayview Circle, Baltimore, MD 21224 USA

**Keywords:** Drug hypersensitivity, *Clostridium difficile* colitis, Vancomycin

## Abstract

**Background:**

Oral vancomycin is a first-line treatment for severe *Clostridium difficile* colitis. Oral vancomycin is perceived to lack systemic absorption or systemic adverse effects; however, a few cases of hypersensitivity to oral vancomycin have been reported, all in hospitalized patients.

**Case presentation:**

In the present case, a 66-year-old woman with end-stage neurodegenerative disease residing in a long-term care facility developed a maculopapular rash following treatment with enteral vancomycin for recurrent *C. difficile* colitis. The rash resolved after withdrawal of the drug.

**Conclusion:**

Rashes associated with oral vancomycin treatment include maculopapular rash, urticaria, red man syndrome, and linear IgA bullous dermatitis. Risk factors for systemic vancomycin absorption include renal insufficiency, severe intestinal inflammation, and high vancomycin dose and duration. Routine serum testing of vancomycin levels, even in these high risk cases, is not recommended. Clinicians should be aware that enteral vancomycin can cause hypersensitivity reactions which may be serious.

## Background

*Clostridium difficile* remains an important infection in long-term care facilities, contributing to morbidity and mortality [1]. Orally administered vancomycin, generally an effective treatment for *C. difficile* colitis, is perceived to lack the risk of systemic adverse drug reactions or hypersensitivity reactions seen with intravenous administration. However, there have been 11 published cases of cutaneous reactions to enteral vancomycin [2–12] (Table 1). We present a case of hypersensitivity to enteral vancomycin in a long-term care facility. The patient’s legal representative provided consent for publication of this case.

**Table 1:** Published cases of reactions to oral vancomycin

Reference	Sex	Age	Reaction	Vancomycin dose, treatment duration before rash	Vancomycin serum level	Risk factors
Bailey et al. [2]	M	82	Red man syndrome	250 mg every 6 h, 4 days	Not done	Acute on chronic kidney disease, high vancomycin dose
Baumgartner et al. [3]	M	51	Maculopapular rash	125 mg every 6 h, 3 days	Not done	Diverticulitis, severe CDI
Bergeron et al. [4]	M	23 months	Red man syndrome	90 mg every 6 h, first day	28.7 mcg/mL	Multiple drug hypersensitivities
Bossé et al. [5]	M	35	Anaphylaxis	500 mg, first dose	Not done	Cystic fibrosis, multiple hypersensitivities-IV vancomycin hypersensitivity, severe CDI
Choudhry et al. [6]	F	60	Linear IgA bullous dermatosis	Unknown dose, 14 days	Not reported	Prolonged duration of treatment
Killian et al. [7]	F	67	Red man syndrome	500 mg every 6 h, first day	4.1 mcg/mL	High vancomycin dose
McCullough et al. [8]	F	82	Maculopapular rash	125 mg every 6 h, 8 days	Not done	Chronic kidney disease
Mizumura et al. [9]	M	76	Maculopapular Rash	500 mg every 6 h, 9 days	3.9 mcg/mL	High vancomycin dose, severe CDI
Nallasivan et al. [10]	M	58	Red man syndrome	Dose not reported, 3 days	Not done	Acute kidney injury requiring dialysis, ICU care
O’Brien et al. [11]	M	45	Linear IgA bullous dermatosis	Unknown dose, 2 days	Not reported	End stage renal failure
Osawa et al. [12]	F	73	Maculopapular rash	250 mg, first day after completing oral desensitization	Not done	IV vancomycin hypersensitivity, recent colon perforation
Current report	F	66	Maculopapular rash	125 mg every 6 h, 4 days	0.1 mcg/mL	

CDI, *Clostridium difficile* infection; d, days; f, female; h, hour; m, Male

## Case presentation

A diabetic, chronically ventilated woman in her late 60 s with end-stage neurodegenerative disease, residing in a long-term care ventilator unit, developed diarrhea that was positive for *C. difficile* toxin 1 month after treatment with a first-generation cephalosporin for cellulitis. She began enteral metronidazole per gastrostomy tube, but diarrhea continued. She was switched to enteral vancomycin. The diarrhea stopped, but recurred 2 weeks later, associated with low-grade fever, leukocytosis, hyperglycemia, and hypoalbuminemia. Enteral vancomycin was restarted (125 mg, four times/day) and diarrhea improved.

On day 4 of treatment, red macules, papules and patches appeared on her thighs, torso, and back. The neck and upper extremities were generally unaffected and there was no mucous membrane involvement. She was afebrile (36.0 °C), her pulse was 95 beats-per-minute (bpm) and blood pressure was 125/70 mmHg. Cardiac and pulmonary examinations were unremarkable. Her abdominal examination showed no organomegaly and there was no change from baseline in the neurological examination. White blood count (WBC) was 7.500/mL with 73% neutrophils, 20% lymphocytes, 5% monocytes, and 2% eosinophils. Renal function tests were subtly elevated (creatinine 0.3 mg/dL vs. 0.16 mg/dL baseline and blood urea nitrogen 6.7 mmol/L [normal range 2.5–6.4 mmol/L]). Her blood glucose level was 255 mg/dL and albumin dropped from 3.0 g/dL to 2.3 g/dL. Gamma-glutamyltransferase (GGT) was 191 IU/L (normal range 5–85 IU/L) alkaline phosphatase 223 IU/L (normal range 46–116 IU/L); and transaminases (aspartate transaminase [AST] 50 IU/L [normal range 15–37 IU/L] and alanine transaminase [ALT] 93 IU/L [normal range 12–78 IU/L]) were slightly elevated. Elevated liver enzymes were not a new finding.

The only changes in treatment in the days preceding the rash were introduction of vancomycin and insulin, which she had received in the past, and a change in the brand of food administered through her gastrostomy tube. Her other medications were ipratropium, zolpidem, trazodone, potassium gluconate, moxifloxacin eye drops, artificial tears, and oxycodone/acetaminophen. After the rash appeared, vancomycin was stopped. Metronidazole was restarted with a dose of 500 mg three times/day and diarrhea slowly abated. Her rash was treated symptomatically with systemic antihistamines (chlorpheniramine), resolving within 3 days. Other medications were continued, including the new tube feeding brand. Her serum vancomycin level when the rash occurred was 0.1 mcg/mL. The rash did not recur and she was not rechallenged with vancomycin because her diarrhea responded to metronidazole. No other patients or staff members had similar rashes. There was no infectious outbreak at the time in the unit. As noted, her rash was not associated with fever and was also not associated with other viral or autoimmune symptoms. According to the Naranjo Adverse drug reaction probability scale [13], this rash was a probable reaction to vancomycin (score 6/13).

## Discussion and conclusions

We present a case of a maculopapular rash in a chronically-ventilated woman with end-stage neurodegenerative disease, residing in a long-term care facility. The rash developed 4 days after initiation of enteral vancomycin treatment for *C. difficile* diarrhea. Several cases of oral vancomycin skin reactions have been reported, all in acute-care hospitals (see Table 1) [2–12], most in patients over age 60.

Reactions have included anaphylaxis, red man syndrome, linear IgA bullous dermatitis, and maculopapular rash. Red man syndrome is characterized by a red rash, predominantly on the head and upper body following vancomycin administration. It is caused by mast cell degranulation rather than by IgE-mediated allergy. Vancomycin-induced linear IgA bullous dermatosis is characterized by tense grouped bullae and caused by IgA deposits at the dermo-epidermal junction [6].

Other than IgA bullous dermatosis, other non-immediate vancomycin hypersensitivity reactions can include DRESS syndrome (drug reaction with eosinophilia and systemic symptoms), acute interstitial nephritis, and Stevens-Johnson syndrome [14]. These other hypersensitivity reactions have not been reported after enteral vancomycin use. Only one case of an IgE-mediated reaction to enteral vancomycin has been reported [5], but it can be difficult to differentiate IgE-mediated hypersensitivity reactions from red man syndrome [14].

Studies have not detected serum vancomycin levels in healthy populations receiving oral vancomycin [15]; however, several studies have identified detectable vancomycin levels in individuals with *C. difficile* infection, presumably reflecting the impact of gastrointestinal tract inflammation. Although enteral administration of vancomycin minimizes systemic absorption, 58 of 85 participants who received enteral vancomycin in one study of patients with *C. difficile* had a detectable serum vancomycin level ≥ 0.05 mcg/mL [16]. The frequency of detectable serum vancomycin levels in patients receiving oral treatment has varied from 2% to 68% in published studies [16–18].

Risk factors for systemic absorption of oral vancomycin in one study include renal insufficiency, severe *C. difficile* (age 65 and above is one criteria for that category, according to some definitions), high vancomycin dose (> 500 mg/day), prolonged therapy (> 10 days), ICU admission, use of vancomycin retention enemas, and GI tract inflammation [16]. Among published cases of oral vancomycin reactions, most patients had at least one of these risk factors. Several patients also had a history of other hypersensitivities or cystic fibrosis, both general risk factors for drug hypersensitivity. In one study, prolonged fasting and massive diarrhea were risk factors for systemic absorption [19].

Systemic vancomycin has been associated with cutaneous adverse reactions. Younger age and prolonged duration of therapy were risk factors for cutaneous adverse reactions in an older study [20], and they accounted for almost half of adverse drug reactions from systemic vancomycin in one report [21].

Because *C. difficile* infections as well as *C. difficile* infection severity both seem to be increasing [22], oral vancomycin will likely be prescribed more frequently and more adverse reactions will be seen.  Nevertheless, routine serum vancomycin testing for patients receiving oral vancomycin is not recommended because serum levels in a toxic range are unlikely to be found.

Rechallenge with a suspected source of a drug rash is helpful to confirm the cause of hypersensitivity. In our patient and in many other clinical cases, rechallenge was not performed because alternative pharmacotherapy was available, safe, and effective; thus the risk–benefit ratio did not justify rechallenge [23].

In cases of cutaneous vancomycin reactions, stopping the drug is the key first step of treatment [24]. In a patient with no systemic reaction and no suggestion of immediate hypersensitivity, treating a maculopapular rash with antihistamines while continuing vancomycin may be a reasonable strategy when no alternative treatments are available. This approach has been used successfully with other anti-microbial agents [25].

Management choices for patients with *C. difficile* infection and an intolerance to oral vancomycin could include vancomycin desensitization (which can utilize intravenous doses [26] or oral administration [27]. A successful case of oral vancomycin desensitization over 5 h to treat severe *C. difficile* colitis has been reported [27]. Another option is the use of other treatments such as metronidazole, fidaxomicin, or fecal transplant [28, 29].

Oral vancomycin is used commonly in clinical practice in hospitals as well as in long-term care facilities, and clinicians should be aware of the possibility of systemic adverse effects, including anaphylaxis.
